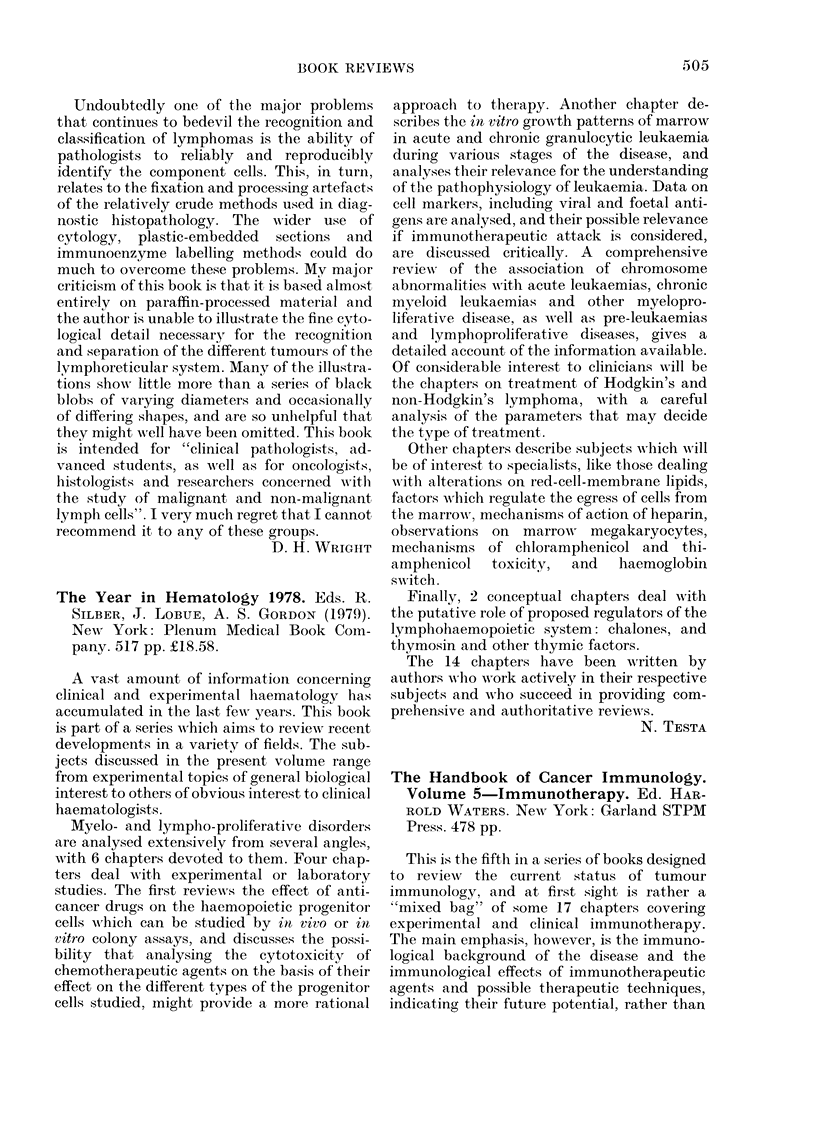# The Year in Hematology 1978

**Published:** 1979-09

**Authors:** N. Testa


**The Year in Hematology 1978.** Eds. R. SILBER, J. LOBUE, A. S. GORDON (1979). New York: Plenum Medical Book Company. 517 pp. £18.58.

A vast amount of information concerning clinical and experimental haematology has accumulated in the last few years. This book is part of a series which aims to review recent developments in a variety of fields. The subjects discussed in the present volume range from experimental topics of general biological interest to others of obvious interest to clinical haematologists.

Myelo- and lympho-proliferative disorders are analysed extensively from several angles, with 6 chapters devoted to them. Four chapters deal with experimental or laboratory studies. The first reviews the effect of anti-cancer drugs on the haemopoietic progenitor cells which can be studied by *in vivo* or *in vitro* colony assays, and discusses the possibility that analysing the cytotoxicity of chemotherapeutic agents on the basis of their effect on the different types of the progenitor cells studied, might provide a more rational approach to therapy. Another chapter describes the *in vitro* growth patterns of marrow in acute and chronic granulocytic leukaemia during various stages of the disease, and analyses their relevance for the understanding of the pathophysiology of leukaemia. Data on cell markers, including viral and foetal antigens are analysed, and their possible relevance if immunotherapeutic attack is considered, are discussed critically. A comprehensive review of the association of chromosome abnormalities with acute leukaemias, chronic myeloid leukaemias and other myeloproliferative disease, as well as pre-leukaemias and lymphoproliferative diseases, gives a detailed account of the information available. Of considerable interest to clinicians will be the chapters on treatment of Hodgkin's and non-Hodgkin's lymphoma, with a careful analysis of the parameters that may decide the type of treatment.

Other chapters describe subjects which will be of interest to specialists, like those dealing with alterations on red-cell-membrane lipids, factors which regulate the egress of cells from the marrow, mechanisms of action of heparin, observations on marrow megakaryocytes, mechanisms of chloramphenicol and thiamphenicol toxicity, and haemoglobin switch.

Finally, 2 conceptual chapters deal with the putative role of proposed regulators of the lymphohaemopoietic system: chalones, and thymosin and other thymic factors.

The 14 chapters have been written by authors who work actively in their respective subjects and who succeed in providing comprehensive and authoritative reviews.

N. TESTA